# Supplementary material for: Waxholm Space atlas of the rat brain: a 3D atlas supporting data analysis and integration
Source: Nat Methods. 2023 Oct 2;20(11):1822–9. doi: 10.1038/s41592-023-02034-3 (PMC10630136; doi:10.1038/s41592-023-02034-3)
Supplement: Supplementary file 1 — Supplementary Table 1 [file 41592_2023_2034_MOESM1_ESM.pdf]

nature methods

Resource

https://doi.org/10.1038/s41592-023-02034-3

Waxholm Space atlas of the rat brain: a 3D atlas supporting data analysis and integration

In the format provided by the authors and unedited

**WHS rat brain atlas v4 label hierarchy**						**Abbreviation**
**1000**	**Brain**					Brain
	**1001**	**White matter**				wmt
		**1050**	**Olfactory white matter**			olf
			180	lateral olfactory tract		lot
		67	corpus callosum and associated subcortical white matter			cc-ec-cing-dwm
		**1002**	**Anterior commissure**			ac
			36	anterior commissure, anterior limb		aca
			37	anterior commissure, posterior limb		acp
			73	anterior commissure, intrabulbar part		aci
		**1003**	**Hippocampal white matter**			hiw
			6	alveus of the hippocampus		alv
			38	ventral hippocampal commissure		vhc
			52	fornix		f
			59	fimbria of the hippocampus		fi
		**1004**	**Corticofugal pathways**			cfp
			1	corticofugal tract and corona radiata		ic-cp-lfp-py
			85	pyramidal decussation		pyx
		**1005**	**Medial lemniscus**			ml
			34	medial lemniscus, unspecified		ml-u
			84	medial lemniscus decussation		mlx
		**1006**	**Thalamic tracts**			tht
			**1051**	**External medullary lamina**		eml
				249	external medullary lamina, unspecified	eml-u
				157	external medullary lamina, auditory radiation	eml-ar
			291	internal medullary lamina		iml
			290	intramedullary thalamic area		ima

			270	superior cerebellar peduncle and prerubral field	scp-pr
			239	pretectothalamic lamina	ptl
			53	mammillotegmental tract	mtg
			54	commissural stria terminalis	cst
			60	fasciculus retroflexus	fr
			61	stria medullaris thalami	sm
			62	stria terminalis	st
			80	habenular commissure	hbc
		63	posterior commissure		pc
		**1007**	**Facial nerve**		7n
			35	facial nerve, unspecified	7n-u
			72	ascending fibers of the facial nerve	asc7
			57	genu of the facial nerve	g7
		**1008**	**Optic fiber system and supraoptic decussation**		ofs
			41	optic nerve	2n
			42	optic tract and optic chiasm	opt-och
			83	supraoptic decussation	sox
		**1009**	**White matter of the tectum**		tew
			46	commissure of the superior colliculus	csc
			68	brachium of the superior colliculus	bsc
			69	inferior colliculus, commissure	cic
			146	inferior colliculus, brachium	bic
		**1010**	**Cerebellar and precerebellar white matter**		cbt
			7	inferior cerebellar peduncle	icp
			78	middle cerebellar peduncle	mcp
			79	transverse fibers of the pons	tfp
		**1011**	**White matter of the brainstem**		bsw
			**1012**	**Lateral lemniscus**	ll

				140	lateral lemniscus, commissure						ll-c
				141	lateral lemniscus, unspecified						ll-u
			129	acoustic striae							as
			130	trapezoid body							tz
			76	spinal trigeminal tract							sp5t
	**1013**	**Gray matter**									GM
		**1052**	**Telencephalon**								**Tel**
				**1053**	**Laminated pallium**						**LamP**
					**1020**	**Olfactory bulb**					OB
						64	Glomerular layer of the accessory olfactory bulb				GlA
						65	Glomerular layer of the olfactory bulb				Gl
						66	Olfactory bulb, unspecified				OB-u
						502	Nucleus of the lateral olfactory tract				NLOT
					**1015**	**Cerebral cortex**					Cx
						**1055**	**Hippocampal region**				HR
							**1021**	**Hippocampal formation**			HF
								99	Fasciola cinereum		FC
								100	Subiculum		SUB
								**1022**	**Cornu Ammonis**		CA
									98	Cornu ammonis 1	CA1
									97	Cornu ammonis 2	CA2
									95	Cornu ammonis 3	CA3
								96	Dentate gyrus		DG
							**1023**	**Parahippocampal region**			PHR
								108	Postrhinal cortex		POR
								109	Presubiculum		PrS
								110	Parasubiculum		PaS
								**1024**	**Perirhinal cortex**		PER
									112	Perirhinal area 35	PER35

									113	Perirhinal area 36	PER36
								**1025**	**Entorhinal cortex**		EC
									114	Medial entorhinal cortex	MEC
									115	Lateral entorhinal cortex	LEC
						**1056**	**Piriform cortex**				PIR
							181	Piriform cortex, layer 1			PIR1
							182	Piriform cortex, layer 2			PIR2
							183	Piriform cortex, layer 3			PIR3
						**1057**	**Cingulate region**				CgR
							**1018**	**Cingulate cortex**			Cg
								411	Cingulate area 1		Cg1
								10	Cingulate area 2		Cg2
							**1058**	**Retrosplenial cortex**			RS
								427	Retrosplenial dysgranular area		RSD
								430	Retrosplenial granular area		RSG
						**1059**	**Insular region**				INS
							**1060**	**Agranular insular cortex**			AI
								409	Agranular insular cortex, ventral area		AI-v
								410	Agranular insular cortex dorsal area		AI-d
								424	Agranular insular cortex, posterior area		AI-p
							414	Dysgranular insular cortex			DI
							416	Granular insular cortex			GI
						**1061**	**Frontal region**				Front
							77	Frontal association cortex			FrA
							**1062**	**Orbitofrontal cortex**			Orb
								403	Medial orbital area		MO
								402	Ventral orbital area		VO
								400	Ventrolateral orbital area		VLO
								401	Lateral orbital area		LO

								404	Dorsolateral orbital area		DLO
							**1063**	**Mediofrontal cortex**			MFC
								405	Prelimbic area		PrL
								413	Infralimbic area		IL
							**1064**	**Motor cortex**			M
								408	Primary motor area		M1
								406	Secondary motor area		M2
								407	Frontal association area 3		Fr3
						**1065**	**Parietal region**				Par
							**1066**	**Somatosensory cortex**			SS
								**1067**	**Primary somatosensory area**		S1
									420	Primary somatosensory area, face representation	S1-f
									425	Primary somatosensory area, barrel field	S1-bf
									418	Primary somatosensory area, dysgranular zone	S1-dz
									417	Primary somatosensory area, forelimb representation	S1-fl
									423	Primary somatosensory area, hindlimb representation	S1-hl
									429	Primary somatosensory area, trunk representation	S1-tr
								422	Secondary somatosensory area		S2
							**1068**	**Posterior parietal cortex**			PPC
								433	Parietal association cortex, medial area		mPPC
								432	Parietal association cortex, lateral area		lPPC
								436	Parietal association cortex, posterior area		PtP
						**1069**	**Occipital region**				Oc
							**1070**	**Visual cortex**			Vis
								442	Primary visual area		V1
								1071	Secondary visual area		V2
									448	Secondary visual area, medial part	V2M
									443	Secondary visual area, lateral part	V2L
						**1072**	**Temporal region**				Te

							444	Temporal association cortex			TeA
							**1019**	**Auditory cortex**			Au
								151	Primary auditory area		Au1
								1073	Secondary auditory area		Au2
									152	Secondary auditory area, dorsal part	Au2-d
									153	Secondary auditory area, ventral part	Au2-v
				**1074**	**Non-laminated pallium**						**N-LamP**
					412	Claustrum					CLA
					500	Endopiriform nucleus					Endo
					501	Amygdaloid area, unspecified					Am-u
				**1075**	**Subpallium**						SubPAL
					**1076**	**Striatum**					Str
						197	Caudate putamen				CPu
						**1077**	**Nucleus accumbens**				NAc
							184	Nucleus accumbens, core			NAc-c
							192	Nucleus accumbens, shell			NAc-sh
						199	Ventral striatal region, unspecified				VSR-u
					**1028**	**Pallidum**					PAL
						**1078**	**Globus pallidus external**				GPe
							195	Globus pallidus external, medial part			GPe-m
							198	Globus pallidus external, lateral part			GPe-l
						32	Entopeduncular nucleus				EP
						193	Ventral pallidum				VP
					**1079**	**Basal forebrain region**					BRF
						82	Basal forebrain region, unspecified				BFR-u
						93	Bed nucleus of the stria terminalis				BNST
						40	Septal region				Sep
						3	Subthalamic nucleus				STh
		**1080**	**Diencephalon**								**Dien**

			**1081**	**Prethalamus**				**Thal-Pre**
				**1033**	**Reticular (pre)thalamic nucleus**			RT
					200	Reticular (pre)thalamic nucleus, unspecified		RT-u
					164	Reticular (pre)thalamic nucleus, auditory segment		RT-a
				**1082**	**Zona incerta**			ZI
					235	Zona incerta, dorsal part		ZI-d
					236	Zona incerta, ventral part		ZI-v
					257	Zona incerta, rostral part		ZI-r
					287	Zona incerta, caudal part		ZI-c
					238	Zona incerta, A13 dopamine cells		ZI-A13
					284	Zona incerta, A11 dopamine cells		ZI-A11
					280	Fields of Forel		FoF
				204	Pregeniculate nucleus			PrG
				281	Subgeniculate nucleus			SubG
				272	Intergeniculate leaflet			IGL
			**1083**	**Epithalamus**				**Thal-EPI**
				206	Lateral habenular nucleus			LHb
				207	Medial habenular nucleus			MHb
				81	Nucleus of the stria medullaris			SMn
				43	Pineal gland			PG
			**1084**	**Dorsal thalamus**				**Thal-D**
				**1085**	**Anterior nuclei of the dorsal thalamus**			ANT
					213	Anterodorsal thalamic nucleus		AD
					**1086**	**Anteroventral thalamic nucleus**		AV
						214	Anteroventral thalamic nucleus, dorsomedial part	AV-dm
						215	Anteroventral thalamic nucleus, ventrolateral part	AV-vl
					254	Anteromedial thalamic nucleus		AM
					255	Interanteromedial thalamic nucleus		IAM
				**1087**	**Dorsal-caudal midline group of the dorsal thalamus**			DC-MID

					242	Paraventricular thalamic nuclei (anterior and posterior)		PV
					260	Intermediodorsal thalamic nucleus		IMD
					211	Parataenial thalamic nucleus		PT
					278	Subparafascicular nucleus		SPF
					208	Posterior intralaminar nucleus		PIL
				**1088**	**Ventral midline group of the dorsal thalamus**			V-MID
					216	Rhomboid thalamic nucleus		Rh
					219	Reuniens thalamic nucleus		Re
					268	Retroreuniens thalamic nucleus		RRe
					218	Xiphoid thalamic nucleus		Xi
				**1089**	**Mediodorsal nucleus of the dorsal thalamus**			MD
					232	Mediodorsal thalamic nucleus, lateral part		MD-l
					233	Mediodorsal thalamic nucleus, central part		MD-c
					240	Mediodorsal thalamic nucleus, medial part		MD-m
				**1090**	**Ventral nuclei of the dorsal thalamus**			VENT
					293	Ventral anterior thalamic nucleus		VA
					221	Ventromedial thalamic nucleus		VM
					231	Ventrolateral thalamic nucleus		VL
					223	Angular thalamic nucleus		Ang
					**1091**	**Ventral posterior thalamic nucleus**		VPN
						227	Ventral posteromedial thalamic nucleus	VPM
						294	Ventral posterolateral thalamic nucleus	VPL
						266	Ventral posterior nucleus of the thalamus, parvicellular part	VP-pc
					222	Submedius thalamic nucleus		SMT
				**1092**	**Intralaminar nuclei of the dorsal thalamus**			ILM
					246	Paracentral thalamic nucleus		PCN
					247	Central medial thalamic nucleus		CM
					248	Central lateral thalamic nucleus		CL
					267	Parafascicular thalamic nucleus		PF

					282	Ethmoid-Limitans nucleus		Eth
				**1093**	**Posterior complex of the dorsal thalamus**			PoC
					230	Posterior thalamic nucleus		Po
					210	Posterior thalamic nuclear group, triangular part		Po-t
				**1094**	**Lateral posterior (pulvinar) complex of the dorsal thalamus**			LP
					285	Lateral posterior thalamic nucleus, mediorostral part		LP-mr
					286	Lateral posterior thalamic nucleus, mediocaudal part		LP-mc
					283	Lateral posterior thalamic nucleus, lateral part		LP-l
				**1095**	**Laterodorsal thalamic nuclei of the dorsal thalamus**			LD
					228	Laterodorsal thalamic nucleus, dorsomedial part		LD-dm
					229	Laterodorsal thalamic nucleus, ventrolateral part		LD-vl
					205	Dorsal lateral geniculate nucleus		DLG
				**1032**	**Medial geniculate complex of the dorsal thalamus**			MG
					298	Medial geniculate body, ventral division		MG-v
					295	Medial geniculate body, dorsal division		MG-d
					150	Medial geniculate body, marginal zone		MG-mz
					297	Medial geniculate body, medial division		MG-m
					299	Medial geniculate body, suprageniculate nucleus		MG-sg
			**1034**	**Hypothalamus**				HY
				48	Hypothalamic region, unspecified			HTh-u
			**1096**	**Pretectum**				PreT
				94	Pretectal region			PRT
				163	Nucleus sagulum			Sag
		**1097**	**Mesencephalon**					Mes
			**1035**	**Midbrain**				MB
				**1036**	**Tectum**			Tc
					**1037**	**Inferior colliculus**		IC
						142	Inferior colliculus, dorsal cortex	DCIC
						143	Inferior colliculus, central nucleus	CNIC

						145	Inferior colliculus, external cortex	ECIC
					**1038**	**Superior colliculus**		Su
						50	Superficial gray layer of the superior colliculus	SuG
						55	Deeper layers of the superior colliculus	SuD
				**1039**	**Tegmentum**			Tg
					**1098**	**Substantia nigra**		SN
						187	Substantia nigra, reticular part	SN-r
						188	Substantia nigra, compact part	SN-c
						189	Substantia nigra, lateral part	SN-l
					196	Ventral tegmental area		VTA
					201	Peripeduncular nucleus		PP
					71	Interpeduncular nucleus		IP
					51	Periaqueductal gray		PAG
			47	Brainstem, unspecified				BS-u
		**1099**	**Rhombencephalon**					**Rho**
			**1100**	**Metencephalon**				Met
				58	Pontine nuclei			Pn
				**1047**	**Cerebellum**			Cb
					4	Molecular cell layer of the cerebellum		Cb-m
					5	Cerebellum, unspecified		Cb-u
			**1101**	**Myelencephalon**				Myel
				**1043**	**Cochlear nucleus, ventral part**			VCN
					158	Ventral cochlear nucleus, anterior part		AVCN
					159	Ventral cochlear nucleus, posterior part		PVCN
					160	Ventral cochlear nucleus, cap area		Cap
					123	Ventral cochlear nucleus, granule cell layer		GCL
				**1044**	**Cochlear nucleus, dorsal part**			DCN
					126	Dorsal cochlear nucleus, molecular layer		DCNM
					127	Dorsal cochlear nucleus, fusiform and granule layer		DCNFG

					128	Dorsal cochlear nucleus, deep core	DCND
				75	Spinal trigeminal nucleus		Sp5n
				56	Periventricular gray		PVG
				**1045**	**Superior olivary complex**		SO
					131	Nucleus of the trapezoid body	NTB
					132	Superior paraolivary nucleus	SPN
					133	Medial superior olive	MSO
					134	Lateral superior olive	LSO
					135	Superior periolivary region	SPR
					136	Ventral periolivary nuclei	VPO
				**1046**	**Nuclei of the lateral lemniscus**		NLL
					137	Lateral lemniscus, ventral nucleus	VLL
					138	Lateral lemniscus, intermediate nucleus	ILL
					139	Lateral lemniscus, dorsal nucleus	DLL
				74	Inferior olive		IO
	**1048**	**Ventricular system**					V
		33	Ventricular system, unspecified				V-u
		125	4th ventricle				4V
		70	Central canal				CC
45	Spinal cord						SpC
**1049**	**Inner ear**						IE
	119	Vestibular apparatus					VeA
	120	Cochlea					Co
	121	Cochlear nerve					8cn
	122	Vestibular nerve					8vn
	162	Spiral ganglion					SpG
