# Supplementary material for: Waxholm Space atlas of the rat brain: a 3D atlas supporting data analysis and integration
Source: Nat Methods. 2023 Oct 2;20(11):1822–9. doi: 10.1038/s41592-023-02034-3 (PMC10630136; doi:10.1038/s41592-023-02034-3)
Supplement: Supplementary file 2 — Reporting Summary [file 41592_2023_2034_MOESM2_ESM.pdf]

nature portfolio

Corresponding author(s): Trygve B. Leergaard

Last updated by author(s): 2023/06/13

Reporting Summary

Nature Portfolio wishes to improve the reproducibility of the work that we publish. This form provides structure for consistency and transparency in reporting. For further information on Nature Portfolio policies, see our Editorial Policies and the Editorial Policy Checklist.

Statistics

For all statistical analyses, confirm that the following items are present in the figure legend, table legend, main text, or Methods section.

n/a	Confirmed	
☐	☒	The exact sample size (*n*) for each experimental group/condition, given as a discrete number and unit of measurement
☒	☐	A statement on whether measurements were taken from distinct samples or whether the same sample was measured repeatedly
☒	☐	The statistical test(s) used AND whether they are one- or two-sided<br>*Only common tests should be described solely by name; describe more complex techniques in the Methods section.*
☒	☐	A description of all covariates tested
☒	☐	A description of any assumptions or corrections, such as tests of normality and adjustment for multiple comparisons
☒	☐	A full description of the statistical parameters including central tendency (e.g. means) or other basic estimates (e.g. regression coefficient) AND variation (e.g. standard deviation) or associated estimates of uncertainty (e.g. confidence intervals)
☒	☐	For null hypothesis testing, the test statistic (e.g. *F*, *t*, *r*) with confidence intervals, effect sizes, degrees of freedom and *P* value noted<br>*Give P values as exact values whenever suitable.*
☒	☐	For Bayesian analysis, information on the choice of priors and Markov chain Monte Carlo settings
☒	☐	For hierarchical and complex designs, identification of the appropriate level for tests and full reporting of outcomes
☒	☐	Estimates of effect sizes (e.g. Cohen's *d*, Pearson's *r*), indicating how they were calculated

Our web collection on statistics for biologists contains articles on many of the points above.

Software and code

Policy information about availability of computer code

Data collection	The ITK-snap tool (version 3.6.0; RRID:SCR_002010) was used to create delineations.
Data analysis	The VisuAlign (v0.9; RRID:SCR_017978) and the Nutil tool (v0.7.0; RRID:SCR_017183) for data analysis.

For manuscripts utilizing custom algorithms or software that are central to the research but not yet described in published literature, software must be made available to editors and reviewers. We strongly encourage code deposition in a community repository (e.g. GitHub). See the Nature Portfolio guidelines for submitting code & software for further information.

Data

Policy information about availability of data

All manuscripts must include a data availability statement. This statement should provide the following information, where applicable:

- Accession codes, unique identifiers, or web links for publicly available datasets
- A description of any restrictions on data availability
- For clinical datasets or third party data, please ensure that the statement adheres to our policy

All data generated or analysed in this study are included in this article or available through the EBRAINS research infrastructure (https://ebrains.eu) and the Neuroimaging Tools and Resources Collaboratory (NITRC).

The new version of the Waxholm Space atlas of the Sprague Dawley rat brain (version 4) is shared on the atlas home page through NITRC (https://www.nitrc.org/

March 2021

projects/whs-sd-atlas/) and consist of three files:
• WHS_SD_rat_atlas_v4.nii.gz; Volumetric atlas file of 222 anatomical structures.
• WHS_SD_rat_atlas_v4.label; Label file specifying the ID, colour code, and name of each anatomical structure.
• WHS_SD_rat_atlas_v4_PMOD.zip; MBAT-ready atlas with label (.ilf) and startup file (.atlas).
An updated version 4.01 with 224 annotations incorporates minor adjustments made in response to reviewer comments.

Data used to aid delineation of new structures in the atlas are available from the EBRAINS Knowledge Graph:
• Histological and immunohistochemical data stained for parvalbumin, calbindin, NeuN, and myelin (https://doi.org/10.25493/AMW1-Z16, https://doi.org/10.25493/JQ8F-TNF, https://doi.org/10.25493/MZDT-WX4, https://doi.org/10.25493/C63A-FEY).
• Spatial co-registration data for the Paxinos and Watson (stereotaxic; https://doi.org/10.25493/XQ8J-TNE), Paxinos and colleagues (MRI-based; https://doi.org/10.25493/9BHD-WDP), and Swanson (https://doi.org/10.25493/486N-966) reference atlases.

Parvalbumin data re-used to exemplify the use of the new atlas in the QUINT workflow are available from the EBRAINS Knowledge Graph (https://doi.org/10.25493/KR92-C33). The derived data generated through our re-use (i.e. the source data for quantitative results in Figure 4) are provided with this paper.

Human research participants

Policy information about studies involving human research participants and Sex and Gender in Research.

Reporting on sex and gender	N/A
Population characteristics	N/A
Recruitment	N/A
Ethics oversight	N/A

Note that full information on the approval of the study protocol must also be provided in the manuscript.

Field-specific reporting

Please select the one below that is the best fit for your research. If you are not sure, read the appropriate sections before making your selection.

☒ Life sciences ☐ Behavioural & social sciences ☐ Ecological, evolutionary & environmental sciences

For a reference copy of the document with all sections, see nature.com/documents/nr-reporting-summary-flat.pdf

Life sciences study design

All studies must disclose on these points even when the disclosure is negative.

Sample size	The current study uses no new animal data, but re-uses data generated in previous studies. Thus, ethical approval for the current study was not required. All data re-used in this study have complied with ethical regulations for animal research, with statements on this available from the relevant publications
Data exclusions	No data was excluded
Replication	Replication is not relevant as this publication presents a resource
Randomization	Randomization was not relevant to the study, as it is based on a single animal and thus no assignation to groups occurred.
Blinding	Blinding was not relevant to the study, as it is based on a single animal.

Reporting for specific materials, systems and methods

We require information from authors about some types of materials, experimental systems and methods used in many studies. Here, indicate whether each material, system or method listed is relevant to your study. If you are not sure if a list item applies to your research, read the appropriate section before selecting a response.

Materials & experimental systems

n/a	Involved in the study
☒	☐ Antibodies
☒	☐ Eukaryotic cell lines
☒	☐ Palaeontology and archaeology
☒	☐ Animals and other organisms
☒	☐ Clinical data
☒	☐ Dual use research of concern

Methods

n/a	Involved in the study
☒	☐ ChIP-seq
☒	☐ Flow cytometry
☒	☐ MRI-based neuroimaging
